# MYSM1/2A-DUB is an epigenetic regulator in human melanoma and contributes to tumor cell growth

**DOI:** 10.18632/oncotarget.18617

**Published:** 2017-06-27

**Authors:** Christina Wilms, Carsten M. Kroeger, Adelheid V. Hainzl, Ishani Banik, Clara Bruno, Ioanna Krikki, Vida Farsam, Meinhard Wlaschek, Martina V. Gatzka

**Affiliations:** ^1^ Department of Dermatology and Allergic Diseases, Ulm University, 89081 Ulm, Germany; ^2^ ETH, 8092 Zurich, Switzerland; ^3^ Department of Neurology, Ulm University, 89081 Ulm, Germany

**Keywords:** epigenetics, melanoma, pigmentation, transcriptional regulation, UV-radiation

## Abstract

Histone modifying enzymes, such as histone deacetylases (HDACs) and polycomb repressive complex (PRC) components, have been implicated in regulating tumor growth, epithelial-mesenchymal transition, tumor stem cell maintenance, or repression of tumor suppressor genes - and may be promising targets for combination therapies of melanoma and other cancers. According to recent findings, the histone H2A deubiquitinase 2A-DUB/Mysm1 interacts with the p53-axis in hematopoiesis and tissue differentiation in mice, in part by modulating DNA-damage responses in stem cell and progenitor compartments. Based on the identification of alterations in skin pigmentation and melanocyte specification in Mysm1-deficient mice, we hypothesized that MYSM1 may be involved in melanoma formation. In human melanoma samples, expression of MYSM1 was increased compared with normal skin melanocytes and nevi and co-localized with melanocyte markers such as Melan-A and c-KIT. Similarly, in melanoma cell lines A375 and SK-MEL-28 and in murine skin, expression of the deubiquitinase was detectable at the mRNA and protein level that was inducible by growth factor signals and UVB exposure, respectively. Upon stable silencing of *MYSM1* in A375 and SK-MEL-28 melanoma cells by lentivirally-mediated shRNA expression, survival and proliferation were significantly reduced in five *MYSM1* shRNA cell lines analyzed compared with control cells. In addition, MYSM1-silenced melanoma cells proliferated less well in softagar assays. In context with our finding that MYSM1 bound to the *c-MET* promoter region in close vicinity to PAX3 in melanoma cells, our data indicate that MYSM1 is an epigenetic regulator of melanoma growth and potentially promising new target for tumor therapy.

## INTRODUCTION

Melanoma – an aggressive malignancy arising from melanocytes with increasing incidence worldwide – is associated with early metastasis, poor response to therapeutic interventions at later stages, and high mortality [1, 2]. In line with the model of multi-step tumorigenesis, alterations in oncogenes, in particular *BRAF* and *N-RAS*, as well as in tumor suppressor (TS) genes, such as Cyclin-dependent kinase inhibitor 2a (*CDKN2a*), Phosphatase and Tensin homolog (*PTEN*) and also TS *p53*, are required for the transformation process of melanocytes to melanoma cells (reviewed in [3]). In addition, epigenetic changes occur in the skin – in particular in response to UV-exposure and upon aging – and often critically contribute to melanoma formation (summarized in [3, 4]). Apart from altered DNA-methylation patterns and microRNA deregulation, histone modifying enzymes such as histone deacetylases (HDAC) and polycomb repressive complex (PRC) members EZH2, JARID2, and BMI1, have been implicated in melanoma growth, epithelial-mesenchymal transition (EMT), and metastasis (reviewed in [4–6]). However, the interplay of the diverse set of transcriptional regulators and signaling cascades at different stages of melanoma formation and tumor recurrence is still only incompletely understood. Despite tremendous improvements in the therapeutic arsenal against metastatic melanoma and other tumors in recent years – with clinical approval of targeted and immunomodulatory therapies including BRAF- and MEK-inhibitors as well as anti-CTLA4 and anti-PD1 antibodies (summarized in [7, 8]) – primary and acquired resistance of tumor cells towards therapy remains a major challenge.

The enzyme Myb-like SWIRM and MPN domains 1 (MYSM1, also called 2A-DUB) belongs to the SWIRM family of chromatin associated factors and catalyzes the removal of ubiquitin from Lysine residue K119 of histone H2A [9]. Early studies have linked H2A deubiquitination to prostate cancer where MYSM1 regulated androgen receptor-dependent gene activation by coordinating histone acetylation and deubiquitination, and destabilizing the association of linker histone H1 with nucleosomes [9]. In Mysm1-deficient mouse models, additional functions of the deubiquitinase in hematopoietic stem cells (HSC), lymphocyte differentiation and mature blood cells could be identified ([10, 11], and others). More recently, Mysm1 has been shown to regulate murine hematopoiesis and tissue development through interplay with tumor suppressor gene *p53* and its target genes [12–14]. In human hematopoiesis, similar functions of MYSM1 may be required because rare inactivating *MYSM1* mutations were associated with inherited bone marrow failure syndromes [15]. Moreover, in genetic screens *Mysm1* has been identified as gene with essential functions in murine skin development [16]. However, the mechanisms linking deubiquitination by Mysm1 to the regulation of normal skin functions and potentially malignant transformation have not been investigated in detail.

Based on our finding that Mysm1-deficient mice have several p53-dependent developmental anomalies, including altered skin structure and pigmentation – in part resembling mouse phenotypes commonly found upon deletion of genes, such as *PAX3*, *MITF*, and *SOX10*, associated with melanoma in humans [17] – we hypothesized that *MYSM1* as well might be involved in tumorigenesis and melanoma. In this investigation, we therefore analyzed the function of MYSM1 in melanocytes and melanoma cells using mouse models, patient material, and tumor cell lines. In addition, we explored how MYSM1 as histone-modifying enzyme may regulate tumor genes in melanoma.

## RESULTS

### Mysm1 regulates skin pigmentation in mice

**Figure 1:**
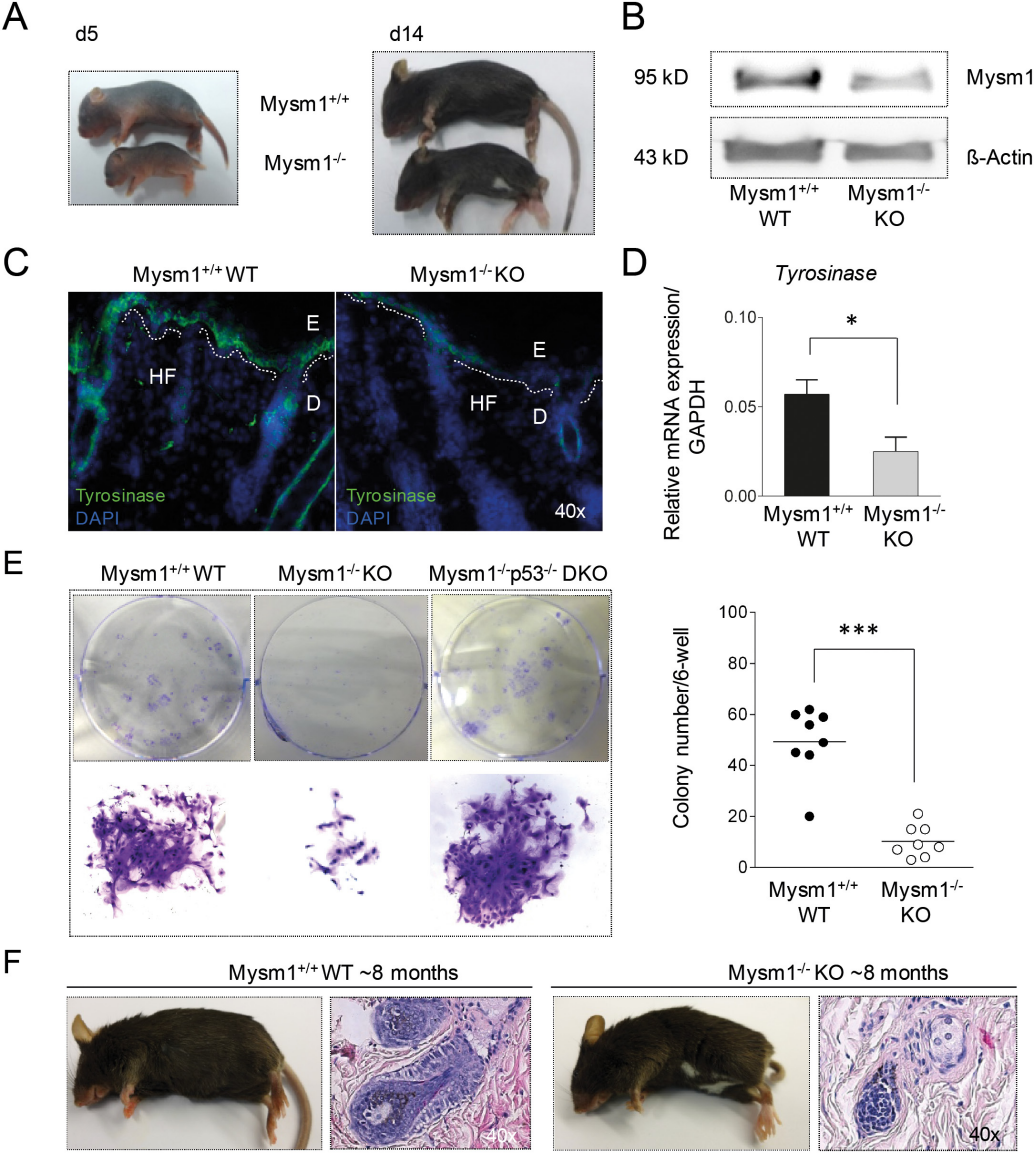
Pigmentation defect and altered melanocyte specification in Mysm1^−/−^ mice **(A)** Postnatal development of visible skin pigmentation in Mysm1^−/−^ (KO) in comparison with wild-type (WT) mice. The belly-spot-and-tail-phenotype manifested around 14 days after birth of Mysm1^−/−^ mice. **(B)** Mysm1 protein expression in murine skin by Western Blot relative to Δ-Actin. Low residual Mysm1 expression in KO mice reflects hypomorphic Mysm1 alleles. **(C)** Tyrosinase expression in newborn Mysm1^−/−^ skin compared with WT littermates (Tyrosinase green, DAPI-stained nuclei blue, dotted white lines separate E:epidermis and D:dermis, HF:hair follicle, n≥3, representative slides shown). **(D)** qPCR analysis of *Tyrosinase* mRNA expression in newborn Mysm1^−/−^ and WT control mice relative to GAPDH. Bar graphs show mean results of 3 independent experiments with at least 3 mice of each genotype, standard deviations as indicated. **(E)** Reduced colony formation potential of precursor cells derived from skin of 8-week-old Mysm1^−/−^ compared with WT and p53^−/−^Mysm1^−/−^ DKO mice under melanocyte differentiation condition (n>4). **(F)** Monitoring of hair graying in adult 6- to 9-month-old Mysm1^−/−^ and WT mice (representative photos shown, n>6 each genotype) and H/E stainings of corresponding skin samples (original magnification 40x).

Visible anomalies of Mysm1-deficient (Mysm1^−/−^ KO) mice included a so-called “belly-spot-and-tail” (*Bst*) phenotype that manifested around 14 days of age (Figure 1A). White belly spots are commonly detectable in mouse strains upon deletion of genes that have regulatory functions in melanocyte differentiation and melanoma [17, 18]. In accordance with potential functions of Mysm1 in the skin, moderate Mysm1 protein expression was detectable in total skin samples from wild-type (WT) mice by Western Blot (Figure 1B) and qPCR (not shown). Confirming a potential role of Mysm1 in murine melanocyte specification, the *Bst*-phenotype correlated with reduced expression of Tyrosinase (Tyr), an enzyme exclusively expressed by melanocytes, in skin sections of newborn Mysm1^−/−^ mice compared with WT littermates (Figure 1C) and qPCR analyses (Figure 1D). In 8-week-old Mysm1^−/−^ skin, the reduction of *Tyr* mRNA was less profound (not shown). However, precursors derived from skin of 8-week-old Mysm1^−/−^ mice had reduced melanocyte colony formation potential *in vitro* as indicated by fewer and smaller colonies under melanocyte differentiation conditions in comparison with WT littermates and p53^−/−^Mysm1^−/−^ mice (Figure 1E). The rescue of Mysm1^−/−^ melanocyte colony formation potential upon simultaneous deletion of p53 may result from increased cellular survival [12] or increased precursor frequencies. In line with reduced differentiation of Mysm1^−/−^ precursors towards melanocytes *in vitro* and grossly normal melanocyte maintenance, 6- to 9-month-old Mysm1^−/−^ mice did not suffer from premature hair graying or increased hair loss upon age compared with age-matched WT littermates (Figure 1F). Because accelerated hair graying is generally regarded as an indicator of premature differentiation or loss of melanocyte stem cells (McSC) as well as altered melanocyte homeostasis in the hair follicle bulge [19, 20], we concluded that in contrast to its function in melanocyte specification, Mysm1 did not seem to be required for McSC maintenance or normal melanocyte homeostasis in adult mice.

### MYSM1 expression is upregulated in human melanoma samples compared with normal human skin

**Figure 2:**
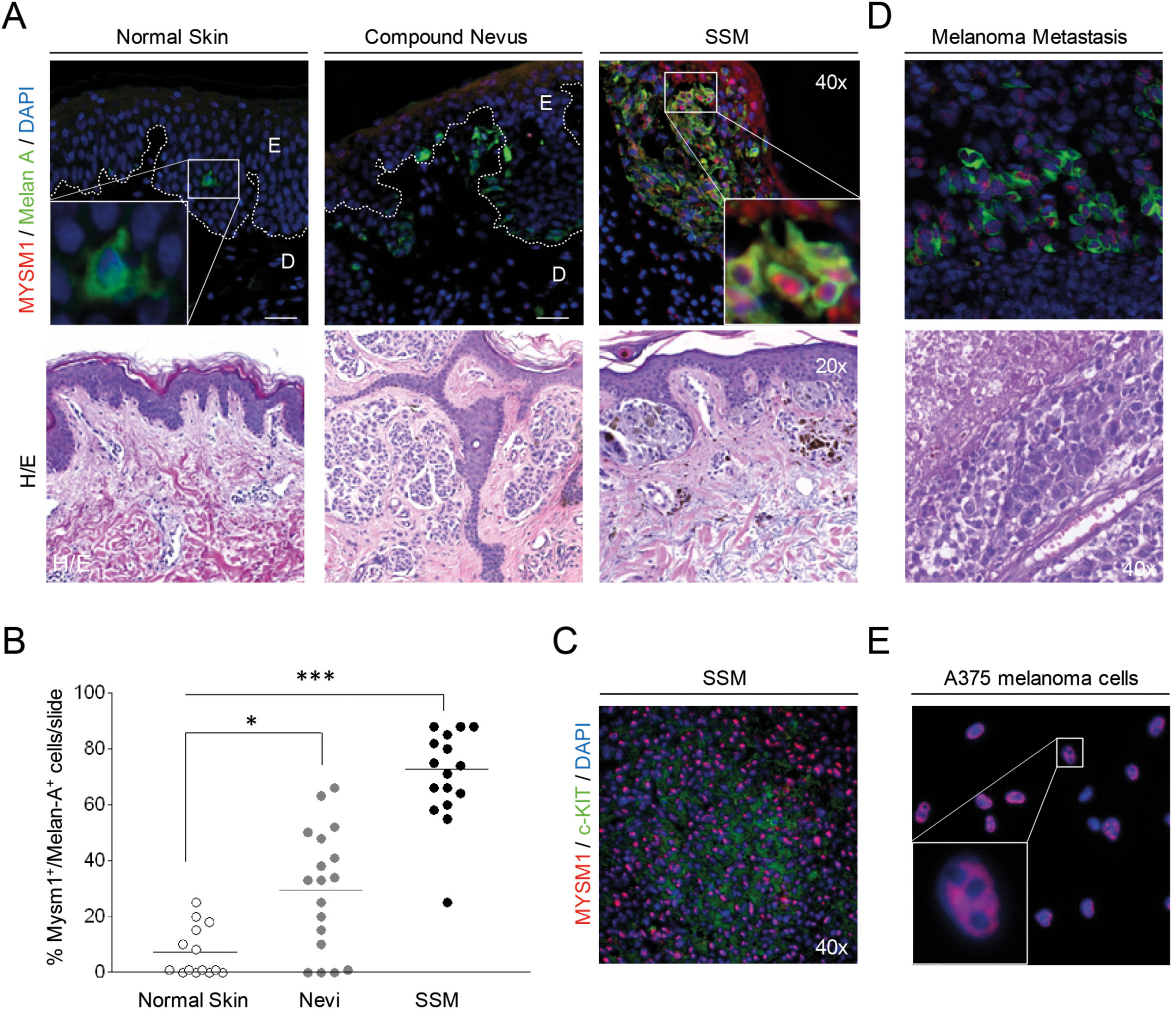
MYSM1 expression in normal human skin, nevi, primary melanoma, and melanoma metastases **(A)** Representative microphotographs of IF analyses of normal skin, dysplastic nevi from 6 patients, or SSM sections from 10 melanoma patients, and corresponding Hematoxylin&Eosin stainings from the same sections. The white dotted lines indicate the borders between epidermis (E) and dermis (D). MYSM1 protein expression was up-regulated in the nuclei of Melan A-positive cells in SSM and to a lesser extent in nevi compared with normal skin (MYSM1 red, Melan-A green, DAPI-stained nuclei in blue, original magnification as indicated). **(B)** Quantification of Melan-A^+^MYSM1^+^ double-positive cells in normal skin, melanocytic nevi (n=6) and SSM (n=10). **(C)** Co-localization of MYSM1 and c-KIT in selected SSM samples (MYSM1 red, c-KIT green, DAPI-stained nuclei blue). **(D)** MYSM1 protein expression in melanoma skin metastases identified by double-staining of MYSM1 and Melan-A (MYSM1 red, Melan-A green, DAPI-stained nuclei blue, representative IF, n=6). **(E)** Localization of MYSM1 protein in A375 melanoma cells in culture (MYSM1 red, DAPI-stained nuclei blue, representative photo, n>6).

Because similar pigmentation phenotypes as observed in Mysm1^−/−^ mice are commonly caused by deletion of genes such as Paired box 3 (*Pax3*), SRY box 10 (*Sox10*), Microphthalmia-associated transcription factor (*Mitf*), and others which human homologs are involved in melanoma formation [17], we hypothesized that MYSM1 may be implicated in *melanomagenesis* as well. To at first analyze expression levels and distribution of MYSM1 in human melanocytes *versus* nevi and melanoma cells *in situ*, paraffin tissue sections of normal human skin and human melanoma samples were subjected to immunofluorescent (IF) staining against MYSM1 in combination with melanocyte marker Melan-A. In normal human melanocytes, negligible or only low levels of MYSM1 protein were detectable as shown by almost absent co-staining of Melan-A-positive cells in normal skin (Figure 2A, first panel). However, significant amounts of MYSM1 were detectable in the nuclei of Melan-A-positive melanoma cells in all 10 human superficial spreading melanoma (SSM) samples tested (Figure 2A, third panel, representative sample). In addition, MYSM1 was expressed in tumor-infiltrating immune cells in the dermis in most SSM samples analyzed. Expression of MYSM1 in melanocytic nevi was less consistent and at an intermediate level compared with normal melanocytes and melanoma (Figure 2A, middle panel). Quantification of MYSM1^+^Melan-A^+^ double-positive cells in normal skin, melanocytic nevi, and melanoma confirmed the increase in MYSM1 expression during step-wise transformation of normal melanocytes to malignant melanoma cells (Figure 2B). Similarly, MYSM1 was found at increased levels in c-KIT-positive melanoma (Figure 2C and Supplementary Figure 1). In melanoma skin metastases, MYSM1 expression was consistently detectable in the majority of tumor cell nuclei as confirmed by double-staining for MYSM1 and Melan-A (Figure 2D). In line with high expression in SSM and melanoma metastasis *in situ*, human melanoma cell lines A375 and SK-MEL-28 were strongly positive for MYSM1 that mainly localized to the nuclei (Figure 2E and Supplementary Figure 2).

### Mysm1 is induced by UVB in murine skin and regulated by growth factor signals in A375 melanoma cells

**Figure 3:**
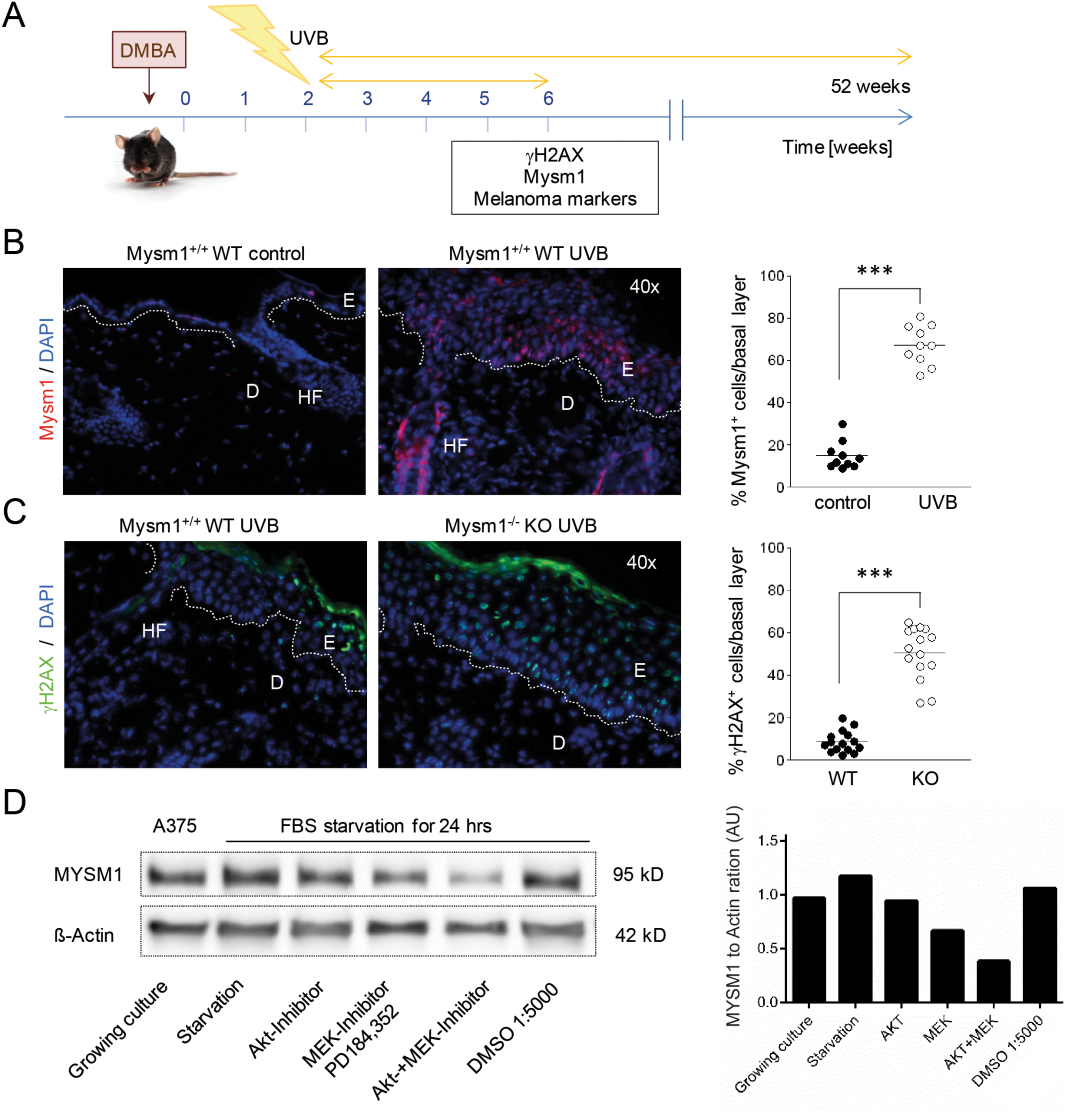
MYSM1 is regulated by UV-exposure of the skin and by growth factor signals in A375 melanoma cells **(A)** Schematic illustration of the UVB-irradiation protocol used to induce DNA-lesions in Mysm1^−/−^ mice and WT littermates. **(B)** Mysm1 expression in mouse skin exposed to UVB over the course of 4 weeks compared with aged-matched untreated skin (Mysm1 red). **(C)** γH2AX foci in basal cells of UVB-treated WT and Mysm1^−/−^ KO mice (γH2AX green, n≥3). For the corresponding quantification of Mysm1- and γH2AX-positive cells, positive cells in the basal cell layer in at least 15 high power fields were counted (n>3). **(D)** Western Blot analysis of MYSM1 protein expression in A375 melanoma cells in culture in response to serum-starvation and treatment with either MEK inhibitor PD184,352 or Akt1/2 kinase inhibitor A6730 (2 μM, both Sigma Aldrich) or both for 24 hrs compared to Δ-Actin.

Given the potential function of Mysm1 in DNA-damage response (DDR) pathways and the critical role of UV light in melanoma cell formation and cellular transformation, we subsequently investigated if Mysm1 was inducible in murine skin upon irradiation with UVB using established protocols [21] (Figure 3A). Strong up-regulation of Mysm1 was detectable in the basal layer of UVB-irradiated skin of all four wild-type mice tested in comparison with the control group by IF analysis (Figure 3B). In addition, we found an increased presence of DNA-damage marker γH2AX in epidermal basal cells after UVB-irradiation of Mysm1^−/−^ compared with age-matched wild-type mice (Figure 3C). Increases in γH2AX foci were detectable in Mysm1^−/−^ melanocytes as well as basal keratinocytes as differentiated by double-staining against γH2AX and melanocyte marker Tyrosinase-related protein 2 (TRP2) (Supplementary Figure 3).

Corresponding Western Blot analyses performed with A375 melanoma cells revealed that MYSM1 protein expression was regulated by growth factor signals as indicated by down-regulation of MYSM1 upon serum-starvation in context with inhibition of MEK- and Akt-signaling. Significant reduction of MYSM1 protein expression was detectable upon treatment of A375 cells with either inhibitor PD128,345, targeting mitogen-activated protein kinase kinase MEK, alone or in combination with an Akt1/2-inhibitor for 24 hrs (Figure 3D). Overall, our expression data from human and murine skin samples and human melanoma cell lines indicated that *MYSM1* might be a gene associated with growth and survival of immature melanocyte precursors and melanoma cells that is inducible by UV and growth factor signals.

### Survival and proliferation of melanoma cells is supported by MYSM1

**Figure 4:**
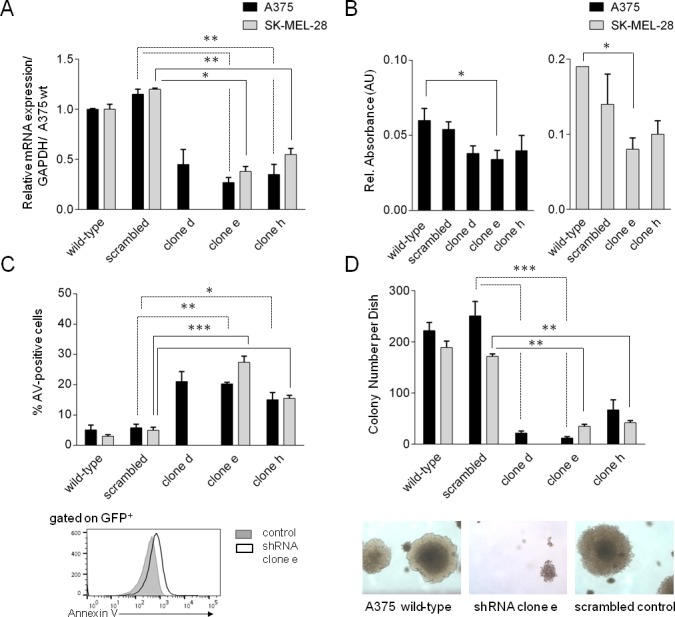
Silencing of MYSM1 in A375 and SK-MEL-28 melanoma cells affects tumor cell survival and proliferation The stable cell lines designated as clones *d*, *e*, and *h* are mixed populations derived from two different parental melanoma cell lines, A375 (black bars) and SK-MEL-28 (grey bars), lentivirally transduced to express *MYSM1* shRNA clones *a-h* and GFP. Scrambled control A375 and SK-MEL-28 cells were stably transduced with scrambled control RNA. Bar graphs and standard deviations in each panel represent results of three independent experiments. **(A)** Knockdown efficiency of *MYSM1* shRNAs in sorted GFP^+^ A375 and SK-MEL-28 melanoma cells compared with scrambled control RNA and parental cells analyzed by FACS and qPCR, respectively. **(B)** MTT assay of melanoma cell viability upon MYSM1 knockdown compared with expression of scrambled control RNA. **(C)** Annexin V-apoptosis assay of GFP-positive melanoma cells after MYSM1 knockdown compared with scrambled control cells by FACS-analysis. **(D)** Softagar-assay of *MYSM1*-silenced in comparison with parental and scrambled control RNA-expressing A375 and SK-MEL-28 melanoma cells. In each case, 10^3^ cells/dish were seeded and colonies with diameter > 125μm were counted after 14 days of incubation.

In order to investigate if MYSM1 – similar to transcriptional regulators like PAX3 or ETS1 – may be critical for melanoma cell survival or proliferation, we subsequently subjected two melanoma cell lines, A375 and SK-MEL-28, to lentiviral transduction with different *MYSM1* shRNA clones designed to stably silence MYSM1 expression. Four shRNA clones generated a significant down-regulation of MYSM1 on the RNA and protein level in the analyzed mixed populations of sorted GFP-positive A375 MYSM1-knockdown cells (termed shRNA clones *d*, *e*, *f* and *h*) as compared with the scrambled control RNA expressing and parental A375 melanoma cells (Figure 4A, Supplementary Figure 4) and were used for further analyses. In addition, two stable SK-MEL-28 MYSM1 knockdown cell lines were established (using shRNA clones *e* and *h*) and analyzed further in comparison with SK-MEL-28 cells expressing scrambled control RNA and wild-type counterparts. Upon *MYSM1* knockdown, reduced overall proliferation and viability of A375 and SK-MEL-28 melanoma cells was measured by trypan blue exclusion and cell counting (not shown). In MTT assays, proliferation of *MYSM1*-silenced A375 cell lines expressing clone *d* and *e* was significantly reduced in comparison with scrambled control and parental A375 cells (Figure 4B, black bars). In addition, increased A375 tumor cell apoptosis upon knockdown of *MYSM1* was detectable by Annexin V staining (Figure 4C, black bars). In SK-MEL-28 cells, MYSM1 knockdown similarly resulted in reduced proliferation and survival (Figure 4B and 4C, grey bars). Subsequently, to test the influence of MYSM1 knockdown on anchorage-independent growth of A375 and SK-MEL-28 melanoma cells, softagar assays were performed as described previously [22]. *MYSM1*-knockdown cell lines *d* and *e* showed significantly reduced colony number and size under anchorage-independent conditions after 14 days of incubation in comparison with scrambled controls and parental A375 as well as SK-MEL-28 cell lines *e* and *h* (Figure 4D).

### MYSM1 binds to the *MET* promoter and co-localizes with PAX3 and c-MET in human melanoma cells

**Figure 5:**
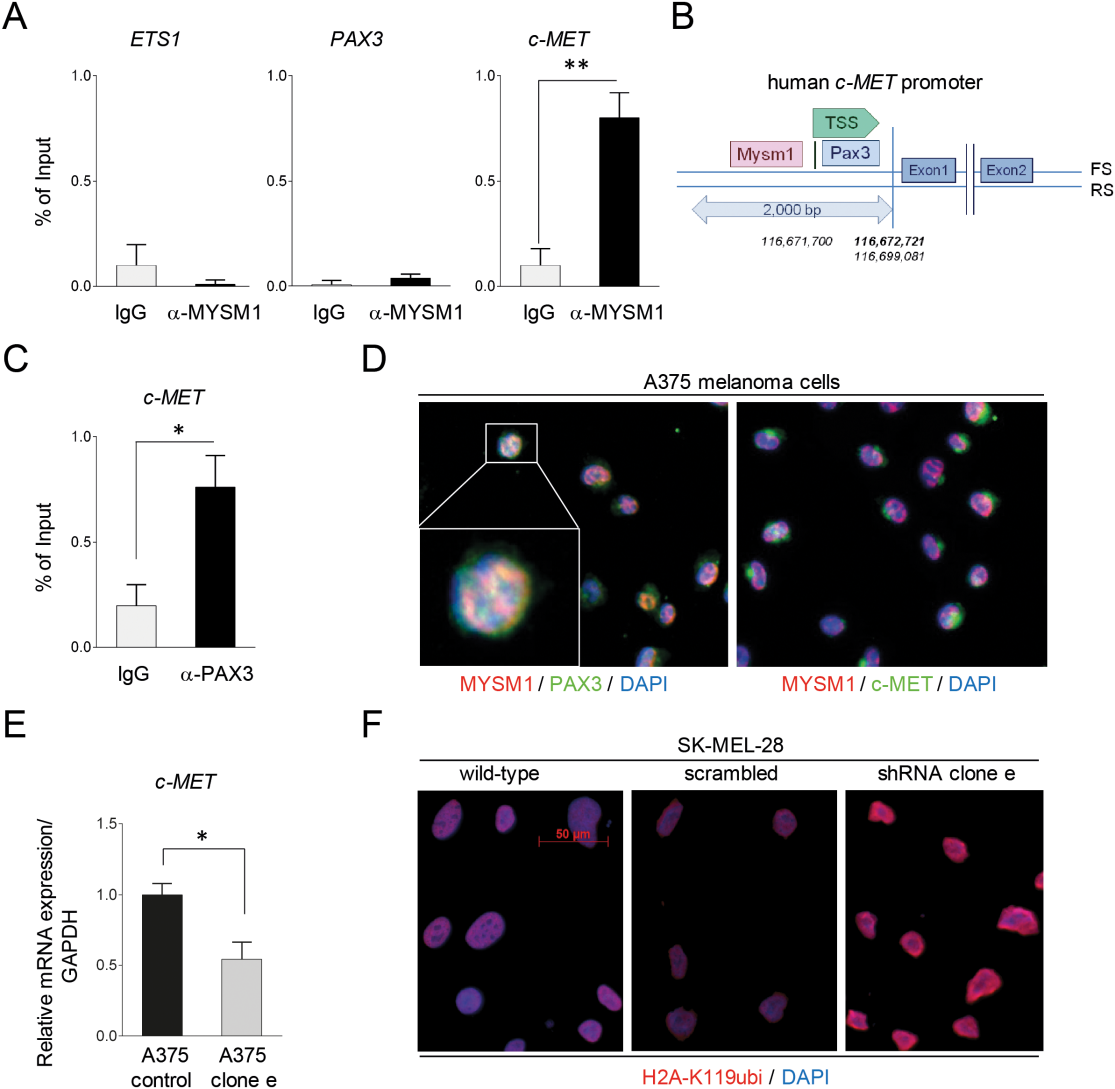
Binding of MYSM1 to the human *c-MET* promoter and co-localization with PAX3 and MET in A375 melanoma cells **(A)** ChIP analysis of MYSM1 binding to a 200 bp fragment close to the TSS of the human *PAX3*, *ETS1*, and *c*-*MET* promoter regions in A375 cells. Bar graphs represent mean values from at least three independent experiments with standard deviations and p-values as indicated. **(B)** Graphical illustration of the localization of the PAX3 consensus DNA binding site and the estimated MYSM1 interaction site in the human *c-MET* promoter region using *BLAST* data. **(C)** Detection of PAX3 binding to an established motive of the c-MET promoter region in A375 cells by ChIP analysis. **(D)** IF analysis of the co-localization of MYSM1 with PAX3 in the nuclei of growing A375 melanoma cells and with c-MET in the cytoplasm (first panel: MYSM1 red, PAX3 green, double-positive areas of nuclear co-localization in yellow; second panel: c-MET green). **(E)** qPCR analysis of *c-MET* mRNA expression in *MYSM1*-silenced in comparison with scrambled control A375 melanoma cells relative to GAPDH (bar graphs represent mean values from at least three independent experiments with standard deviations as indicated). **(F)** IF analysis of SK-MEL-28 wild-type and MYSM1 knockdown melanoma cells (expressing shRNA cl. *e*) with an antibody against H2A-K119ubi (in red, representative images).

As potential mode of function, Mysm1 has been shown to interact with sequence-specific transcription factors (TF) at target gene promoters and to activate gene transcription via its H2A deubiquitinase activity [9–14]. - However, the exact mechanisms of these interactions are still under investigation. Accordingly, MYSM1 may co-operate with TF important for melanoma transformation to promote survival and growth of these tumor cells. Because TFs such as PAX3 and ETS1 have previously been identified to be important regulators in melanoma in part by activating expression of receptor tyrosine kinase c-MET [23], we subsequently performed ChIP assays of the *PAX3*, *ETS* and *c-MET* promoter region using an anti-MYSM1 antibody and chromatin derived from A375 cells. No significant binding of MYSM1 to either the *PAX3* or the *ETS1* promoter sequence around the transcriptional start site (TSS) could be detected in comparison to IgG controls. However, strong MYSM1-binding to a fragment of the *c-MET* promoter close to the TSS was detectable in A375 melanoma cells (Figure 5A). Closer analysis of the *c-MET* promoter region revealed that the site of MYSM1 interaction was adjacent to an established PAX3 binding site [24], likely indicating that MYSM1 action may facilitate PAX3 function at this promoter (Figure 5B). In addition, binding of PAX3 to the established motive in the *c-MET* promoter could be verified by ChIP in A375 cells (Figure 5C). In addition, co-localization of MYSM1 and PAX3 could be detected in the nuclei of exponentially growing A375 melanoma cells by IF staining (Figure 5D and Supplementary Figure 5) – underpinning that these two transcriptional regulators co-operate in gene regulation in melanoma. In line with a function of MYSM1 in the regulation of *c-MET* in melanoma cells, *c-MET* mRNA expression was significantly decreased in A375 cells lentivirally silenced for *MYSM1* in comparison with scrambled control A375 cells (Figure 5E). Moreover, in accord with the function of MYSM1 as H2A deubiquitinase, overall H2A-K119ubi was increased in the nuclei upon knockdown of MYSM1 in A375 and SK-MEL-28 melanoma cells (Figure 5F).

### MYSM1 co-localizes with PAX3 and MET in human SSM samples

**Figure 6:**
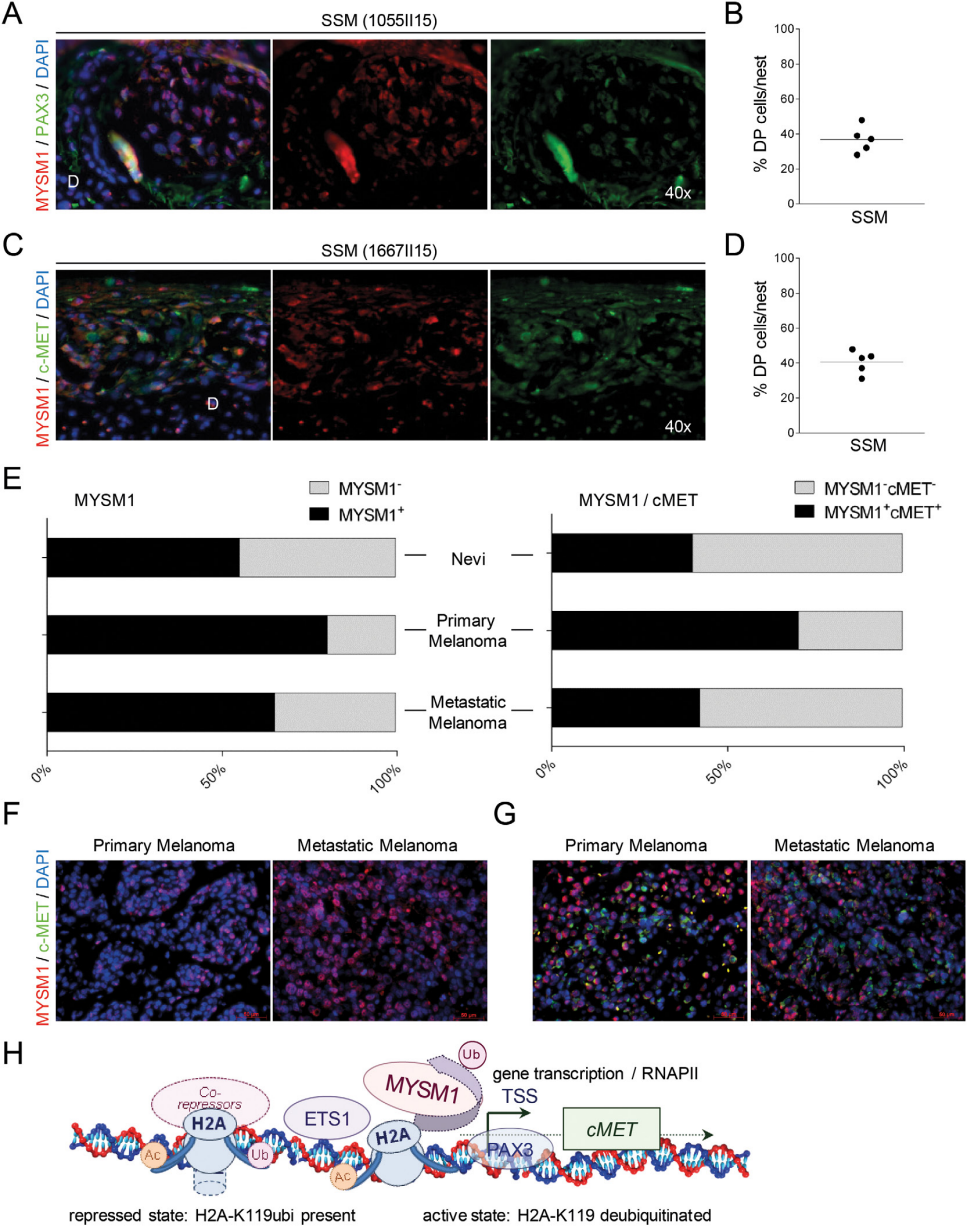
Co-localization of MYSM1 with PAX3 and c-MET in human SSM samples **(A-D)** Representative microphotographs of IF analyses of SSM sections from at least 5 different melanoma patients. For orientation, position of the dermis (D) is indicated. **(A)** Nuclear co-localization of MYSM1 with PAX3 in human melanoma samples (MYSM1 red, PAX3 green, DAPI-stained nuclei in blue, co-localization areas yellow, original magnification 40X). **(B)** To quantify MYSM1^+^, PAX3^+^ and MYSM1^+^PAX3^+^ double-positive cells in SSM samples, positively stained cells in these three categories were counted. For all measurements, the median of specifically stained cells counted in at least 15 high-power fields is presented (sample size n>3). **(C)** MYSM1-expressing cells in SSM sections are often positive for c-MET in the cytoplasma (MYSM1 red, MET green, DAPI-stained nuclei in blue). **(D)** Single- and double-positive cells for MYSM1 and MET were counted and medians calculated according to B. **(E)** Evaluation of MYSM1 expression and co-expression with c-MET in a melanoma tissue microarray (TMA, US Biomax ME1004e) with 82 melanoma cases and 18 nevi cases by IF staining with an antibody against MYSM1 as well as c-MET. Bar graphs show the percentages of primary and metastatic melanoma as well as nevi samples with significant MYSM1 detection (right panel) and with MYSM1 and c-MET co-expression (left panel). **(F)** Representative IF images of MYSM1 expression in cutaneous melanoma and in lymph node metastases. **(G)** IF analysis of co-expression of MYSM1 and c-MET in representative TMA samples (MYSM1 in red, c-MET in green, DAPI-stained nuclei in blue). **(H)** Proposed simplified model of MYSM1 function as co-factor regulating *c-MET* transcription in melanoma cells.

To confirm the potential co-operation of MYSM1 and PAX3 in the regulation of *c-MET* in melanoma cells *in situ*, subsequently, IF analyses of human SSM samples for these three factors were performed. Consistent with the co-localization of MYSM1 and PAX3 in A375 cells *in vitro*, in SSM patient samples analyzed, MYSM1^+^PAX3^+^ double-positive cells could be found preferably in the tumor cell clusters formed by melanoma cells at the epidermal-dermal junction and in the dermis in the majority of SSM samples analyzed (Figure 6A and 6B). In addition, the SSM samples analyzed showed significant co-localization of MYSM1 with tyrosine kinase c-MET in tumor cell clusters (Figure 6C and 6D). A fraction of melanoma cells in these clusters was positive for proliferation marker Ki-67 (Supplementary Figure 5).

In order to validate the concept of MYSM1 upregulation in primary and metastatic melanoma in a larger number of melanoma patients, we subsequently evaluated MYSM1 expression in a melanoma tissue microarray containing 62 malignant melanoma samples, 20 metastatic melanoma, and 18 nevus tissues. According to the data analyses, nuclear MYSM1 was detectable in the majority of malignant primary and metastatic melanoma cases (Figure 6E, right panel and Figure 6F). In addition, in a subfraction of primary and metastatic melanomas, co-expression of MYSM1 with c-MET could be verified (Figure 6E left panel and Figure 6G). These data further confirm the potential relevance of the proposed function of MYSM1 as transcriptional co-regulator in patient tumors where the H2A deubiquitinase may be involved in activating transcription of tumor related genes (Figure 6H).

## DISCUSSION

In the present investigation, we for the first time showed how phenotypical alterations in mice deficient in H2A deubiquitinase 2A-DUB/Mysm1 translate into functions of this enzyme in human tumor cells, using the example of melanoma. Precisely, melanocyte specification in Mysm1-deficient mice was dysfunctional, and accordingly, MYSM1 knockdown in human melanoma cells resulted in reduced cell survival and proliferation. In context with our previous finding that Mysm1 interacts with the p53-axis during development [12], this enzyme may therefore function as critical epigenetic regulator in tumor formation.

Different functional properties of MYSM1 may contribute to the promotion of melanoma cell survival, proliferation, and dedifferentiation - such as (1) positive transcriptional effects within oncogenic signaling cascades, (2) interaction with tumor suppressor and DNA repair pathways, and (3) direct or indirect influence on tumor cell invasiveness, - and potentially other regulatory functions. Firstly, our data indicate that MYSM1 participates in the regulation of genes involved in melanoma survival and proliferation, such as *c-MET*, via functional interaction with the sequence-specific TF PAX3. Both, c-MET as well as PAX3, have previously been implicated in melanoma cell survival [23–26]. In support of a collaboration of MYSM1 with PAX3 in transcriptional regulation, MYSM1 binding to the *c-MET* promoter in melanoma cells occurred in vicinity to the PAX3 consensus sequence previously described [24]. Moreover, MYSM1 was highly expressed and co-localized with PAX3 and c-MET in melanoma cells in culture and in SSM samples *in situ*. Because H2A deubiquitination has previously been shown to generate a permissive environment for recruitment of sequence-specific TFs to their binding sites within promoters [9–11], we propose that during cellular transformation, increased presence of MYSM1 in the nucleus may promote general gene transcription involved in cell growth and proliferation. In support of this concept, 2A-DUB/MYSM1 was originally discovered as regulator of androgen receptor-dependent gene expression in a co-regulatory complex with histone acetylase p/CAF in prostate cancer cells that displayed reduced overall levels of H2A-K119ubi compared with normal prostate tissue [9]. In melanoma, monoubiquitinated H2A-K119 has previously been implicated in RNF2-mediated metastatic behavior through silencing of the *LTBP2* promoter, whereas the growth-promoting potential of PCR1 factor RNF2 was independent of its E3 ubiquitin ligase activity via an interaction with histone acetylase p300 [27]. Because TF involved in melanocyte specification - like PAX3 and SOX10 - are often re-expressed during melanoma formation [17, 28], MYSM1 - in its potential function as co-regulator of gene transcription - may in a similar manner have dual roles early in physiological melanocyte differentiation and pathologically, during melanoma formation. Interestingly, we observed neurological anomalies of Mysm1-deficient mice, including paralysis of the hind limbs and reduced grip force (not shown), that resemble defects caused by deletion of Pax3 [23].

Secondly, MYSM1 may be involved in suppressing the p53-axis during transformation – as melanoma cells often retain functional p53 until the stage of invasive growth and metastasis [2]. In gene array analyses, high MYSM1 levels were frequently detectable in BRAF^V600E^-positive melanoma [29, and biogps.org], where functional suppression of p53-effects may be required. In this context, RNF2 and also PAX3 – apart from growth-promoting effects – have been shown to suppress p53 [30, 31]. In addition, we showed here that Mysm1 is up-regulated upon UVB-exposure in the skin indicating that this enzyme might be involved in the resolution of DNA damage, potentially in concert with other epigenetic factors [32]. Accordingly, in Mysm1^−/−^ skin, γH2AX foci were increased upon UVB-irradiation in comparison to WT skin. During melanoma dedifferentiation, high expression of DNA repair genes correlated with bad prognosis and adoption of metastatic properties [33]. This was attributed to a need for unhindered replication with fast recovery of stalled replication forks in response to spontaneous blockage or induced DNA lesions in primary melanoma cells that will give rise to metastases – resulting in increased resistance to chemo- and radio-therapy on the way [33].

Thirdly, histone-modifying enzymes of the PRC families have been shown to have key roles in the switch of melanoma cells between proliferative and invasive state [4, 27]. Accordingly, inactivation of histone-lysine N-methyltransferase EZH2 with a preclinical inhibitor impaired proliferation and invasiveness accompanied by re-expression of tumor suppressor genes in melanoma cells [34]. Moreover, PCR1 component BMI1 was shown to contribute to the induction of melanoma gene signatures correlating with metastasis as well as chemo-resistance to BRAF-inhibitors [35]. In this context, the dynamic regulation of BMI1 during DNA double-strand break (DSB) repair and its temporary co-localization with γH2AX foci [36, 37] – potentially required to preserve overall genomic integrity in different types of cancer cells – may imply a need for an interaction with MYSM1 or other deubiquitination enzymes to adapt the mono-ubiquitination status of H2A to re-activate transcription. Differential effects of stage- and target gene-specific epigenetic mechanisms and non-canonical functions are underlined by the dual role of PcG factor RNF2 in melanoma proliferation *versus* invasiveness and its interaction with p53 in other tumors [27, 31].

For future studies, it will therefore be interesting to further unravel the exact regulatory mechanisms of *MYSM1* induction during melanocyte transformation to melanoma and melanoma metastases as well as to further investigate the interplay with factors commonly altered in melanoma such as BRAF, N-RAS, PTEN, CDKN2A, p53, and others. In particular, the differential functions of MYSM1 in tumor cells vs. normal (non-transformed) cells expressing MYSM1, such as different types of immune cells, will need to be analyzed in greater detail in order to determine if targeting MYSM1 in combination regimens may hold promise for future therapies of stage IV melanoma patients [38]. Given the roles of MYSM1 and other histone modifiers in promotion of transcription, cellular growth, and transformation [39], it seems likely that functions of 2A-DUB/MYSM1 are essential in a number of tumor types.

## MATERIALS AND METHODS

### Mouse models

Mysm1^tm1a(Komp)Wtsi^ mice (Mysm1^−/−^) have been described previously [10, 12] and were handled in accordance with the guidelines for animal experimentation approved by the Regierungspräsidium Tübingen, Germany.

### Cell culture

A375 and SK-MEL-28 melanoma cells were obtained from the ATCC and cultured in monolayer in DMEM supplemented with 10% fetal bovine serum, L-glutamine, and penicillin/streptomycin as described previously. To test cell viability and proliferation, MTT assays were performed according to the manufacturer's instructions (Cayman Chemicals, Ann Arbor, MI). Annexin V apoptosis assays were conducted as previously described [12]. For primary murine melanocyte colony formation assays under melanocyte differentiation conditions, melanocyte basal medium (PromoCell) was used supplemented with phorbol myristate acetate (PMA), bovine pituitary extract, basic fibroblast growth factor, insulin and hydrocortisone.

### Patient samples

All patient samples were acquired after informed consent according to the Declaration of Helsinki.

### Immunofluorescent analyses

Paraffin-embedded or cryo-preserved tissue samples were processed and stained as previously described [12] and analyzed using a Zeiss AxioImager microscope. For tissue culture IF analyses, parental and shRNA transduced melanoma cell lines were seeded on poly-L-lysine coated slides and subsequently fixed, permeabilized and stained as described in [12]. Specific antibodies against Tyrosinase, MYSM1 (HPA054291, Sigma Aldrich/Atlas Antibodies), Melan-A, c-Kit, PAX3, c-MET, H2A-K119ubi, and γH2AX as well as secondary antibodies (all either from Abcam, Sigma-Aldrich, or DAKO) were used as indicated. Isotype IgG served as negative control in all experiments. Nuclei were visualized by DAPI staining. Original magnification was 40x and representative slides of at least 3 independent samples are shown unless indicated otherwise.

### qPCR

Total RNA was prepared, reversely transcribed, and analyzed by qRT-PCR as previously described [12], and expression levels of target genes were calculated by normalization to GAPDH mRNA expression.

### UVB irradiation

Mice were first treated with 7,12-Dimethylbenz(a)anthracene (DMBA) on depilated back skin for 14 days and then irradiated with 180 mJ/cm^2^ UVB every 3 days for 4 weeks following an established protocol [21] prior to analysis of skin sections.

### shRNA and lentiviral transduction

shRNA clones against human *MYSM1* and scrambled controls were obtained from Genecopoeia (Rockville, MA). Lentiviral Transduction was performed using the plasmids pMD2.G (envelope), pMDLg/pRRE (gag/pol), and pRSV-REV and packing cell line 293T as previously described [22, 40]. Subsequently, melanoma cells expressing either MYSM1 shRNA or scrambled control RNA were purified by FACS based on their co-expression of green fluorescent protein (GFP).

### Softagar assays

The ability of *MYSM1*-knockdown A375 cell lines to grow in anchorage-independence was assessed by colony formation in softagar after 14 days of incubation as described before [22]. Colonies with a diameter > 125 μm were scored as positive.

### ChIP assays

ChIP assays were performed with an α-MYMS1 antibody (orb137033, Biorbyt, Berkeley, CA) using the SimpleChIP® Kit (Cell Signaling, Danvers, MA) according to the manufacturer's instructions as described before.

## SUPPLEMENTARY FIGURES



## Abbreviations

CDKN: Cyclin-dependent kinase inhibitor
DDR: DNA-damage response
DSB: double-strand break
EMT: epithelial-mesenchymal-transition
HDAC: histone deacetylase
IF: immunofluorescence
McSC: melanocyte stem cell
MM: malignant melanoma
MYSM1: Myb-like SWIRM- and MNP-domain containing
PcG: Polycomb Group
PRC: Polycomb repressive complex
SSM: superficial spreading melanoma
TF: transcription factor
TSS: transcriptional start site
TS: tumor suppressor
Tyr: Tyrosinase
UV: ultraviolet
WT: wild-type
