# Supplementary material for: MYSM1/2A-DUB is an epigenetic regulator in human melanoma and contributes to tumor cell growth
Source: Oncotarget. 2017 Jun 27;8(40):67287–99. doi: 10.18632/oncotarget.18617 (PMC5620173; doi:10.18632/oncotarget.18617)
Supplement: Supplementary file 1 [file oncotarget-08-67287-s001.pdf]

MYSM1/2A-DUB is an epigenetic regulator in human melanoma and contributes to tumor cell growth

SUPPLEMENTARY FIGURES

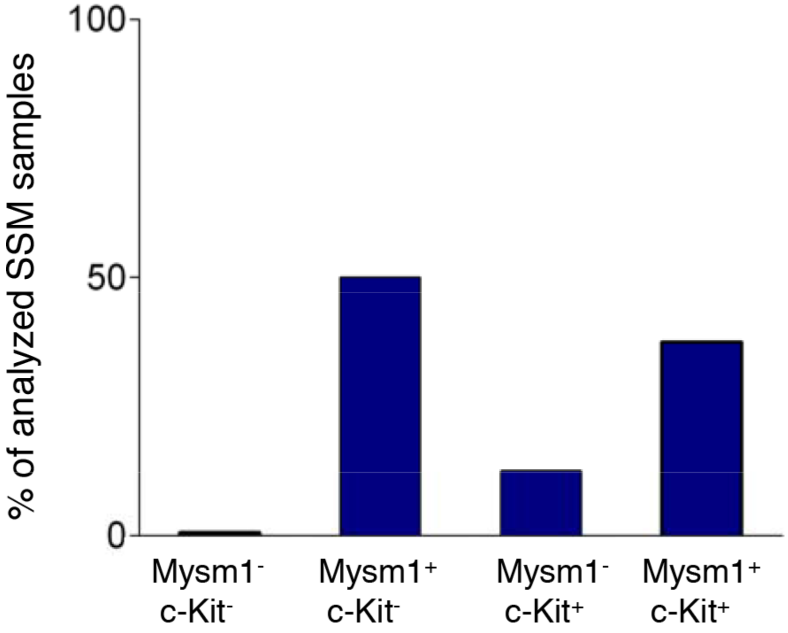

Supplementary Figure 1: Co-expression of MYSM1 and c-KIT in human SSM samples. Graphical illustration of the percentages of analyzed SSM samples containing MYSM1^{+}c-KIT^{+} double-positive cell in clusters *versus* SSM samples with either only MYSM1 or c-KIT expression (total n=8).

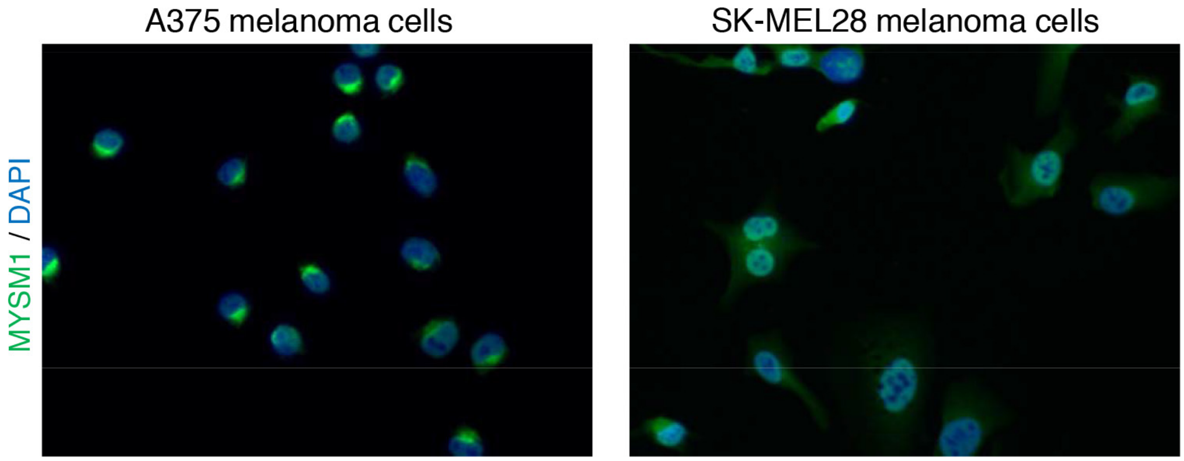

Supplementary Figure 2: MYSM1 protein expression and localization in A375 and SK-MEL-28 melanoma cells. MYSM1 localization in the nucleus of the majority of A375 and SK-MEL-28 melanoma cells. (MYSM1 in green, DAPI-stained nuclei blue, original magnification 40x, n>6, representative photos, not edited).

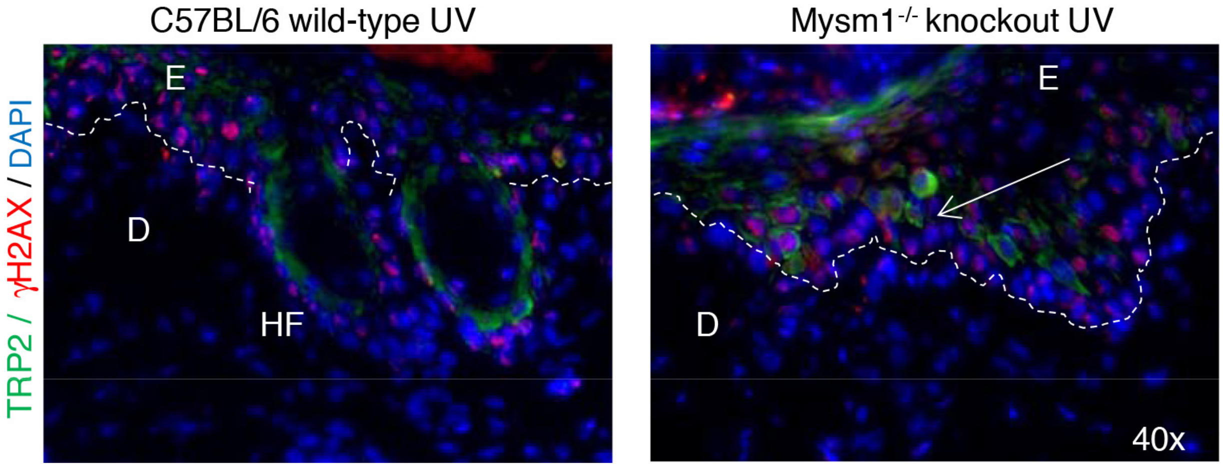

Supplementary Figure 3: Analysis of cell types in the basal layer of UV-irradiated mouse skin. Cryosections of murine skin from either C57BL/6 wild-type or Mysm1$^{tm1a/tm1a}$ (MKO) mice UV-irradiated as described in Figure 3 were subjected to IF analysis with antibodies against gH2AX (red) und Tyrosine-related protein 2 (TRP2, in green). Despite some unspecific staining in the upper epidermis, individual cells could be identified as gH2AX$^+$TRP2$^+$ double-positive melanocytes (white arrow) whereas the majority of gH2AX positive cells were basal keratinocytes (white dotted line shows the border of E:epidermis and D:dermis, HF: hair follicle, original magnification 40x, n≥3, selected images).

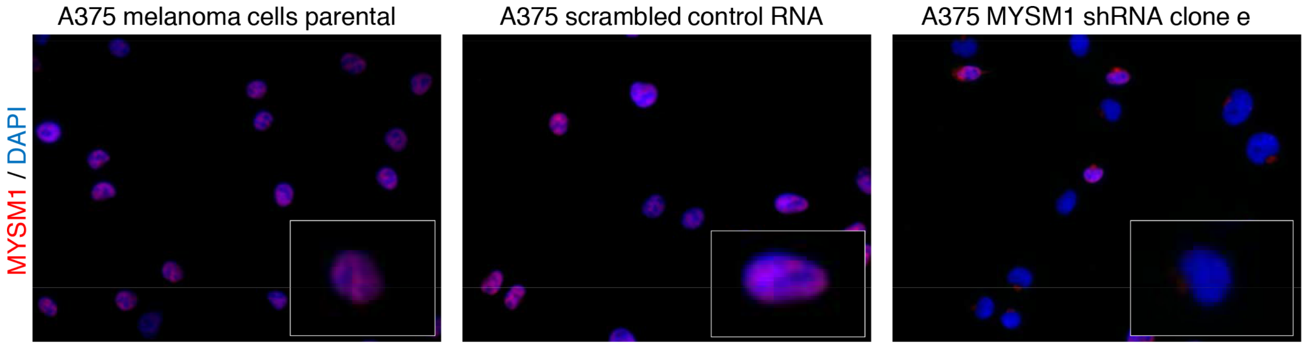

Supplementary Figure 4: Knockdown of MYSM1 protein expression in A375 cells expressing *MYSM1* shRNA clones. MYSM1 in red, DAPI-stained nuclei blue (original magnification 40x, n=8, representative images).

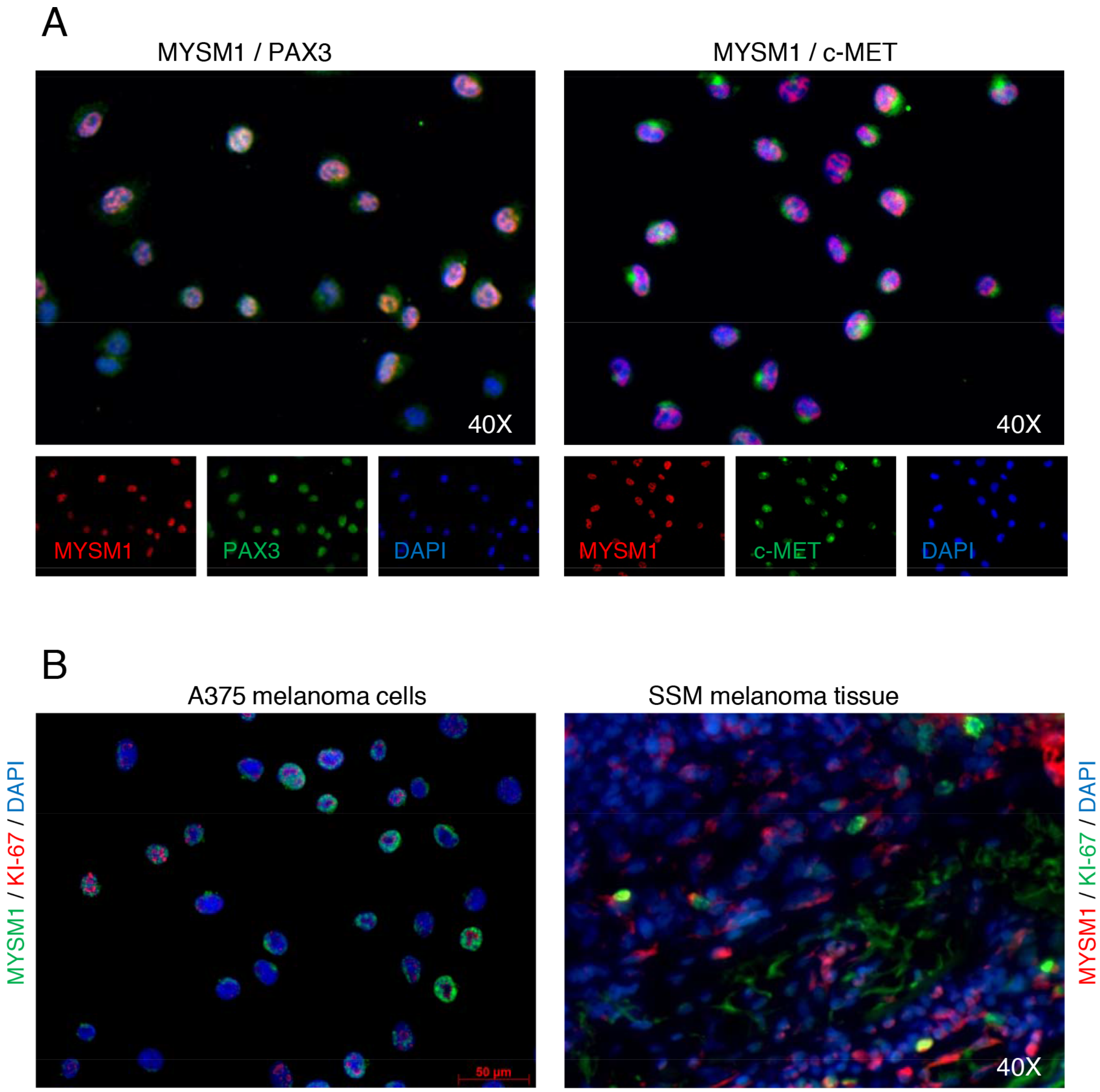

Supplementary Figure 5: Co-localization of MYSM1 with PAX3 and c-MET in A375 melanoma cells and co-expression with Ki-67. **(A)** IF analysis of co-localization of MYSM1 with PAX3 (right panel) and c-MET (left panel) in growing A375 cells (MYSM1 in red, PAX3 or c-MET in green, DAPI-stained nuclei blue). **(B)** Co-expression of MYSM1 and proliferation marker Ki-67 in A375 melanoma cells (right panel) and for comparison in SSM tissue (left panel). (MYSM1 and Ki-67 in green or red as indicated next to the images, DAPI-stained nuclei blue). In A. and B.: original magnification 40x, n>6, representative photos shown.
